# Repurposing of Some Nucleoside Analogs Targeting Some Key Proteins of the Avian H5N1 Clade 2.3.4.4b to Combat the Circulating HPAI in Birds: An In Silico Approach

**DOI:** 10.3390/v17070972

**Published:** 2025-07-10

**Authors:** Mohd Yasir Khan, Abid Ullah Shah, Nithyadevi Duraisamy, Mohammed Cherkaoui, Maged Gomaa Hemida

**Affiliations:** 1 Department of Computer Science, College of Digital Engineering and Artificial Intelligence, Long Island University, Brooklyn, NY 11201, USA; mohd.yasirkhan@liu.edu (M.Y.K.); nithyadevi.duraisamy@liu.edu (N.D.); mohammed.cherkaoui@liu.edu (M.C.); 2 Department of Veterinary Biomedical Sciences, College of Veterinary Medicine, Long Island University, 720 Northern Boulevard, Brookville, NY 11548, USA; abidullah.shah@liu.edu

**Keywords:** highly pathogenic avian influenza virus, H5N1, clade 2.3.4.4b, molecular docking, homology model, hydrogen bond monitor, nucleoside analog

## Abstract

(1) Background: The highly pathogenic avian influenza virus H5N1 clade 2.3.4.4b is an emerging threat that poses a great risk to the poultry industry. A few human cases have been linked to the infection with this clade in many parts of the world, including the USA. Unfortunately, there are no specific vaccines or antiviral drugs that could help prevent and treat the infection caused by this virus in birds. Our major objective is to identify/repurpose some (novel/known) antiviral compounds that may inhibit viral replication by targeting some key viral proteins. (2) Methods: We used state-of-the-art machine learning tools such as molecular docking and MD-simulation methods from Biovia Discovery Studio (v24.1.0.321712). The key target proteins such as hemagglutinin (HA), neuraminidase (NA), Matrix-2 protein (M2), and the cap-binding domain of PB2 (PB2/CBD) homology models were validated through structural assessment via DOPE scores, Ramachandran plots, and Verify-3D metrics, ensuring reliable structural representations, confirming their reliability for subsequent in silico approaches. These approaches include molecular docking followed by molecular dynamics simulation for 50 nanoseconds (ns), highlighting the structural stability and compactness of the docked complexes. (3) Results: Molecular docking revealed strong binding affinities for both sofosbuvir and GS441524, particularly with the NA and PB2/CBD protein targets. Among them, GS441524 exhibited superior interaction scores and a greater number of hydrogen bonds with key functional residues of NA and PB2/CBD. The MM-GBSA binding free energy calculations further supported these findings, as GS441524 displayed more favorable binding energies compared to several known standard inhibitors, including F0045S for HA, Zanamivir for NA, Rimantadine and Amantadine for M2, and PB2-39 for PB2/CBD. Additionally, 50 ns molecular dynamics simulations highlighted the structural stability and compactness of the GS441524-PB2/CBD complex, further supporting its potential as a promising antiviral candidate. Furthermore, hydrogen bond monitor analysis over the 50 ns simulation confirmed persistent and specific interactions between the ligand and proteins, suggesting that GS441524 may effectively inhibit the NA, and PB2/CBD might potentially disrupt PB2-mediated RNA synthesis. (4) Conclusions: Our findings are consistent with previous evidence supporting the antiviral activity of certain nucleoside analog inhibitors, including GS441524, against various coronaviruses. These results further support the potential repurposing of GS441524 as a promising therapeutic candidate against H5N1 avian influenza clade 2.3.4.4b. However, further functional studies are required to validate these in silico predictions and support the inhibitory action of GS441524 against the targeted proteins of H5N1, specifically clade 2.3.4.4b.

## 1. Introduction

**Figure 1:**
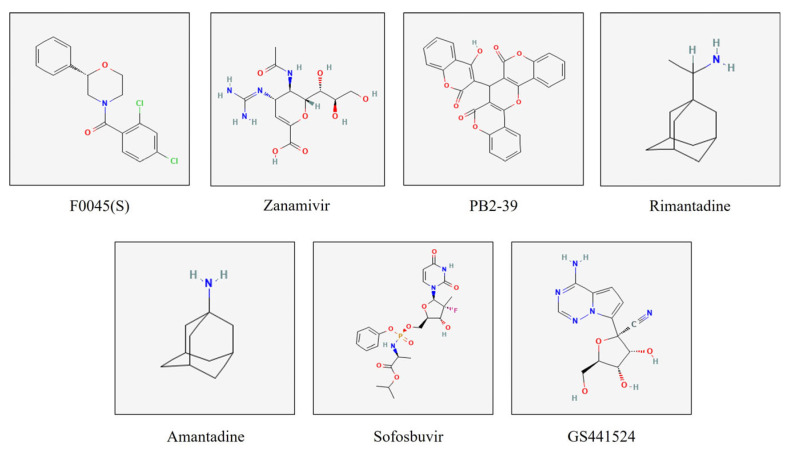
The 2D chemical structures of the tested compounds, sofosbuvir and GS441524, along with the standard inhibitors used in this study. These include F0045(S) (standard for HA protein), Zanamivir (standard for NA protein), PB2-39 (standard for the PB2 cap-binding domain), Rimantadine and Amantadine (standard for M2 protein).

Influenza virus type A (IVA) is an RNA, negative-sense virus with a segmented genome. The viral genome consists of 7–8 segments [1]. Each viral segment encodes at least one protein. These viral proteins include the hemagglutinin (HA), neuraminidase (NA), the matrix protein 2 (M2), the viral nucleoprotein (NP), the viral polymerase called RNA dependent RNA polymerase (RdRp), which consists of three subunits (PA, PB1, and PB2), as well as the nonstructural protein-1 (NSP-1) [1]. Influenza viruses have a unique feature during their transcription. The viral genome is translocated into the host cell nucleus to acquire the cap from the host cell’s DNA polymerase, which primes the viral transcription [2]. The avian influenza virus (AIV) belongs to the influenza virus type A. They have been classified based on their virulence into low-pathogenic (LPAIV) and highly pathogenic (HPAIV) viruses [3]. The influenza viruses are experiencing dynamic changes at the genomic level. There are several reasons for the emergence/reemergence of new viruses, strains, clades, or subclades of avian influenza viruses, including (1) some point mutations across different proteins called antigenic drift, the exchange of some viral genome segments in case of double infection with more than one type of virus called antigenic shift, and (3) the poor proofreading capabilities of the viral RdRp [4,5]. The IVA is classified based on its key proteins into H1-H16 and N1-N9 [6]. This pattern changes frequently in the IVA on the genomic level. These frequent changes of the virus at the genomic level may generate new viral isolates that are resistant to the currently used drugs and vaccines. This is targeting the currently used vaccine and antiviral therapy that is prepared against the previously identified viruses. Thus, there is an urgent need to monitor these viruses at the genomic level through active, vigilant molecular surveillance, as well as the frequent upgrading of vaccines and drugs that match the most recently circulating strains of the virus. The highly pathogenic avian influenza virus continues to pose a great risk to human health and the poultry industry worldwide. During 2021, the H5N1 clade 2.3.4.4b began to cause several outbreaks in poultry farms worldwide. It spread rapidly to different parts of the world [7]. In early 2024, this clade was reported in cattle and milk from many cattle herds across the USA [8]. Human infections due to the same clade have been reported in many countries worldwide [9]. Unfortunately, there is currently no vaccine or antiviral drug specific to prevent or treat infected hosts with this newly emerged clade of HPAIV. Several antiviral drugs were used in the past to treat many IVA in humans. Several strategies can be employed to target various key IAV proteins using these antiviral drugs [10,11,12,13]. Most of these strategies primarily target key viral proteins involved in viral replication and maturation, as well as other host proteins that play important roles during viral replication in host cells [14]. Here, we focus on some antiviral strategies that involve the AIV proteins. These strategies primarily focus on inhibiting viral entry, release, or the processing and maturation of viral virions [14]. Some of these strategies primarily depend on the generation of monoclonal antibodies that neutralize the activities of viral proteins expressed on the surface of the viral envelope, particularly HA and NA [15,16]. In this study, we are mainly focusing on the synthetic antiviral compounds that target various key proteins of the virus. These strategies include (1) inhibitors of HA maturation, (2) inhibitors of the NA protein, (3) inhibitors of RdRp, (4) ion channel (M2) blockers, and (5) inhibitors of NSP1 [14]. There are some compounds that inhibit the maturation of the HA protein of the IAV, such as Nitazoxanide [17]. The NA protein plays crucial roles in AIV replication, including the degradation of mucous layers in the respiratory tract, thereby granting the virus access to epithelial cells. The transmembrane protein NA also plays a crucial role in removing sialic acid from the HA protein. Thus, targeting the NA protein of the AIV is one of the most promising antiviral strategies. Several drugs are currently FDA-approved for targeting the NA protein of AIV, including Zanamivir, Oseltamivir, Peramivir, and Laninamivir [18,19,20]. The viral polymerase RdRp is a promising target for certain antiviral drugs. Several antiviral compounds were shown to inhibit various subunits of this viral polymerase [21,22]. Several compounds targeting the cap-binding domain of the PB2 subdomain (PB2/CBD) have shown some inhibitory effects on viral replication, such as the Pimodivir [23,24]. The nucleoside analogue antiviral drugs, such as Ribavirin, showed a broad-spectrum antiviral activity against many RNA viruses, including IAV [25]. The GS441524, as a nucleoside analog, has been recently used as a powerful inhibitor of the feline infectious peritonitis virus main protease (Mpro) but was never tested as an anti-influenza virus potential drug [26,27]. In the current study, we are comparing the potential impacts of the currently known anti-AIV drugs with some new potential drugs using the molecular docking and simulation approach. The structures of test compounds sofosbuvir, GS441524, and the known standard inhibitors, including F0045(S) for HA, Zanamivir for NA, Rimantadine and Amantadine for M2, and PB2-39 for PB2/CBD, are illustrated in Figure 1. We believe these computational tools will pave the way for screening synthetic and natural compounds to be used as new drugs against many viral diseases, including the currently circulating H5N1 clade 2.3.4.4b. However, further laboratory validations are required to confirm this prediction and to identify the mechanism of action of these potential drugs. To benchmark the performance of test compounds, several well-characterized standard inhibitors were included: F0045(S) for HA, Zanamivir for NA, Rimantadine and Amantadine for M2, and PB2-39 for PB2/CBD. These compounds have previously demonstrated antiviral activity against influenza targets and served as standard ligands in our docking and energy analyses. Their binding affinities, evaluated using MM-GBSA and –CDOCKER scoring functions, were as follows: F0045(S) (−48.86 kcal/mol; 22.92), Zanamivir (−201.00 kcal/mol; 49.26), PB2-39 (−236.80 kcal/mol; 54.55), Rimantadine (−112.23 kcal/mol; 33.02), and Amantadine (−104.46 kcal/mol; 27.55). These values provided a comparative framework for assessing the antiviral potential of the test compounds GS441524 and sofosbuvir (Table 2). The results from our molecular docking and molecular dynamics simulation study suggest that GS441524 exhibits strong binding affinity and dynamic stability with NA and PB2/CBD, indicating its potential for dual inhibition of viral entry and replication. Its broad-spectrum antiviral profile and ability to target conserved influenza domains support its repurpose against H5N1 clade 2.3.4.4b. Experimental validation and combination therapy studies are recommended to further explore its clinical potential.

## 2. Materials and Methods

### 2.1. The H5N1 Clade 2.3.4.4b Sequences Retrieval from the GenBank

In this study, we retrieved the full-length genome sequences of 279 isolates representing the H5N1 clade 2.3.4.4b from the GenBank (NCBI) (https://www.ncbi.nlm.nih.gov/protein, accessed on 15 April 2025) database. The sequence of each protein was downloaded in a fasta format. The sequences of these isolates included (115 from chickens, 40 from ducks, 30 from migratory birds, and 51 from the Canadian geese). We extracted the nucleotides and protein sequences of the HA, NA, PB2/CBD, and M2 proteins from these sequences.

### 2.2. Multiple Sequence Alignment (MSA) and Generation of the Consensus Sequences

We performed the multiple protein sequence alignments using the Biovia Discovery Studio software v24.1.0.321712. The consensus sequences per protein were generated based on the individual’s MSA, which was one per protein. The generated consensus sequences will be used for each protein’s downstream prediction and homology modeling.

### 2.3. The Homology Modeling of the H5N1 Clade 2.3.4.4b (HA, NA, PB2/CBD and M2) Proteins

We used the MODELLER tool of the Biovia Discovery Studio (v24.1.0.321712) and applied the comparative modeling approach to build the homology model for each target protein. This approach enabled the creation of the 3D model structure for the query protein sequences, as previously described [28]. Briefly, the sequences of the target viral proteins HA, NA, and the cap-binding domain of PB2 (PB2/CBD) of the viral RNA polymerase complex RdRp were retrieved and downloaded in FASTA format from the NCBI database (https://www.ncbi.nlm.nih.gov/protein, accessed on 15 April 2025). The homology modeling procedure requires aligning the query sequences with template protein sequences obtained from the BLAST search tool from Biovia Discovery Studio software v24.1.0.321712. The query protein sequence aligned with 100 most identical template sequences with a maximum similarity of 99% and a minimum of 30% in BLAST with the BLOSSUM62 algorithm protocol. Five identical template sequences (similarity: maximum 99–minimum 35%) were chosen to align sequences to the templates. Further, the 3D-build homology model protocol was run to align template structures and predict the query sequence 3D confirmation based on template proteins. The Discrete Optimized Protein Energy (DOPE) score was used to assess the quality of the different 3D protein models generated to predict five different homology models per query sequence. The DOPE score and the PDF total energy score are used to evaluate the accuracy of each protein model. The more negative values of the DOPE score and the more positive PDF total energy values, the more stable and accurate the predicted protein model. The best model, having the lowest DOPE score, was selected for further in silico computational studies as previously described [29]. All the predicted 3D structure models were verified through the Ramachandran plot and the Verify-3D model tool of Biovia Discovery Studio. The Ramachandran plot suggested the stability of structure based on amino acid residues located in the most favored, highly allowed, and allowed regions or sites of the plot. If the verified score results from the Verify-3D method of the modeled protein is higher than the verified expected low score value, then the model is considered of acceptable quality. The closer the verified score result is to the verified expected high score value, the better the quality of the predicted protein structure model. The prediction of structure is further visualized using the Biovia Discovery Studio v24.1.0.321712.

### 2.4. Protein and Ligand Preparation

The prepared proteins, through homology modeling, are used as receptors for ligand interaction. Compounds that are used as ligands in this study are retrieved in 3D-SDF file format from the PubChem database: https://pubchem.ncbi.nlm.nih.gov, accessed on 20 April 2025. For energy minimization of the modeled protein, the smart minimizer at 10,000 steps was run to minimize the energy of the prepared protein through the CHARMm force field. Further, the preparation of the ligand was performed following the energy minimization through the full minimization protocol through smart Minimizer at 10,000 steps with the CHARMm force field using the Biovia Discovery Studio v24.1.0.321712, as previously described [28].

### 2.5. Molecular Docking of Compounds with Target Proteins

The molecular docking approach can be used to model the interaction between HA, NA, and PB2/CBD proteins with some selected and standard anti-influenza virus compounds. The interactions between these small molecules and proteins at the atomic level, molecular docking, help us understand how these molecules bind and function within biological processes [30,31]. The interactions between these small molecules and proteins at the atomic level, molecular docking through the CDOCKER method, help us understand how these molecules bind to target proteins [31,32,33]. Before docking ligands to target proteins, a receptor grid was generated around the binding cavity of the energy-minimized protein molecule by specifying the key amino acid residues. The selected standard ligand of the respective protein molecules was selected to define the amino acid residues for predicting the binding sites. For the generated receptor grid boxes, the binding site spheres were set with x, y, z dimensions and radius for HA (−24.900732 8.841194 53.999225; 12.91), NA (−65.326117 −26.296469 −0.744619; 5.69), PB2/CBD (174.146307 180.457782 137.636677; 10.86), and M2 (0.657379 0.772586 5.639653; 5.0) protein, respectively. Followed by the CDOCKER docking method, we typically use 10 docking poses for the ligand. To consider the best ligand pose out of 10 poses, the higher positive values of the CDOCKER interaction energy score (-CDOCK score) are used. These scores represent the best-bound ligand conformations and relative binding affinities. The computational tool of the Biovia Discovery Studio also enables the visualization of the ligand–target interaction (molecular docking) and the identification of the compounds that bind more efficiently with the target [31]. This analysis typically involves examining the docking scores, ligand–protein interactions, and the visualization of the docked complexes. The docking figures were also created with the BIOVIA Discovery Studio v24.1.0.321712, and the docking scores were ranked, with the most positive scores for each complex chosen. The docking analysis typically involves examining the docking scores, ligand–protein interactions (hydrogen bonds), and the visualization of the docked complexes. To describe the defining binding site in the protein, the interaction binding affinity score (-CDOCKER interaction energy score) is likely an internal step within Biovia Discovery Studio v24.1.0.321712.

### 2.6. Calculations of the Molecular Mechanics-Generalized Surface Area (MM-GBSA)

The binding free energy of the protein–ligand complexes in this study was measured using the MM-GBSA approach, which integrates the molecular mechanics (MM) force fields with a generalized Born and surface area continuum with a non-implicit solvation model. The MM-GBSA calculation incorporates the CHARMm force field, with partial charge estimation using Memory Rone. The dielectric constant was set to be one, and the minimum non-bonded higher and lower cutoff distance was set to be 12 and 10 Å. The MM-GBSA was determined in this study using the equation (ΔG = E_complex (minimized)– [E_ligand (minimized) + E_receptor (minimized)) using the Biovia Discovery Studio v24.1.0.321712. The default setting employed to compute MM-GBSA involved rendering all protein atoms rigid while the ligand atoms are relaxed through the Biovia Discovery Studio software v24.1.0.321712.

### 2.7. Molecular Dynamics (MD) Simulation

Based on in vitro analysis, docking energies, and conformational pose analysis, the top four complexes were selected for MD simulation as previously described [34]. To further explore the dynamic behavior of ligand–protein complexes in this study, the best predicted top hits of compounds and standards with respect to HA, NA, and PB2/CBD, as shown in Table 2, were selected to perform a 50,000 picoseconds (ps) through standard dynamics cascade simulation. To generate the molecular topology files for the protein complex and to create the topology of ligands, the CHARMm36 force field was used. The simulation system consists of an explicit boundary solvent model, an orthorhombic box with a minimal distance of 7 nm between the protein surface and the edge of the box, neutralized with the inclusion of cation type sodium (Na) and anion type Chloride (Cl) counter ions.

For the energy minimization, the steepest descent was (minimization 1) with RMS gradient one, and the conjugate gradient (minimization 2) with RMS gradient 0.1. Both minimization algorithms were used for 50.00 steps. The heating phase was performed using a simulation time of 10 ps with a time step of 2 femtoseconds (fs); for immersion, the initial temperature was 50 K and the target temperature was 300 K, with a save results interval of 2 ps.

The equilibration of complexes was carried out for a 10,000 ps simulation run with a two fs (femtoseconds) time step, and the save result interval is two ps. The Particle–Mesh–Ewald (PME) algorithm was used for long-range electrostatic interactions with fourth-order cubic interpolation and a kappa of 0.34 Å grid spacing. The advanced dynamic integrator used the Leapfrog Verlet algorithm with applied shake constraint. The implicit solvent model was used with a dielectric constant of 1, a nonbond list radius cutoff of 14, in which the nonbond higher cutoff distance is 12 and the nonbond lower cutoff distance is 10. The production step of a standard dynamic cascade of MD simulation was carried out for 50 ns (50000 picoseconds). Trajectory analysis was performed to confirm the number of hydrogen bonds established per each confirmation, hydrogen bond distance variation of ligand and interacted residues per confirmation, Root Mean Square Deviation (RMSD), Root Mean Square Fluctuation (RMSF), and Radius of Gyration (Rg) of each system [35]. The stability of the complex is indicated by the highest potential inhibitor from the stable complex protein–ligand through Biovia Discovery Studio software v24.1.0.321712.

## 3. Results

### 3.1. Homology Modeling of Some Key Proteins of the Avian H5N1 Clade 2.3.4.4b (HA, NA, PB2/CBD, and M2)

**Figure 2:**
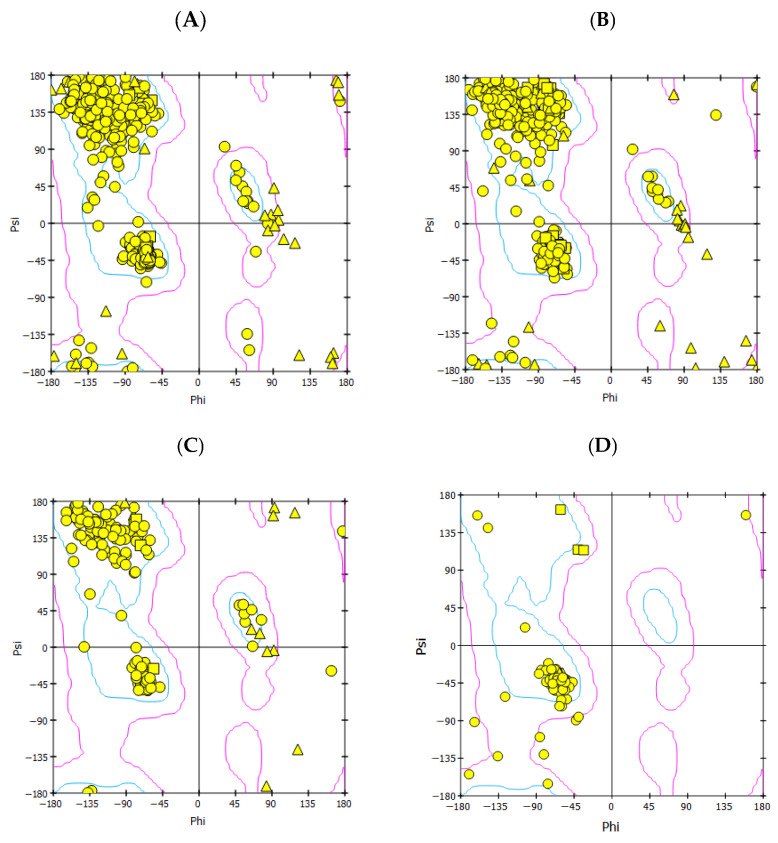
Results of the Ramachandran plot analysis of the predicted homology models of the H5N1 clade 2.2.4.4b proteins (**A**) HA, (**B**) NA, (**C**) PB2/CBD, and (**D**) M2.

To perform the homology modeling, we searched for experimentally determined template reference sequences closely related to the query sequences and shared at least 35–65% sequence identity and similarity. The predicted homology models of HA, NA, PB2/CBD, and M2 proteins were superimposed with their respective template structures used for model generation (Figure S1). Additionally, multiple sequence alignments of HA, NA, M2, and PB2/CBD with their corresponding template sequences revealed conserved regions between the query and template proteins, supporting the structural reliability of the models. These alignments and conserved regions, along with the associated template PDB IDs, are shown in Figures S2–S5. The sequences of the target proteins from H5N1 as receptors are HA (804 AA.), NA (797 AA), PB2/CBD (173 AA) of viral RdRp, and four chain M2 (24 AA each chain) channels. Generally, the predicted homology models, with lower negative DOPE scores and lower positive PDF total energy scores, are considered more structurally stable and reliable (Figure S6). However, if the total energy values for multiple models are very similar, the DOPE score can be used as the deciding factor based on its statistical potential. Therefore, out of five, we selected the best models for each protein based on the lowest total PDF energy values and the lowest DOPE score for each protein homology model, as shown in Table 1. Out of these five predicted homology models, based on the lowest DOPE score and lower positive PDF total energy, one of the best models, which has been further considered to verify the score of predicted 3D protein models, suggests structural integrity and stability (Table 1). The Ramachandran plot results show that the maximum of the total amino acid residues is in the most favored regions, and then is in the additionally most allowed areas. A small proportion of residues are in the generously allowed regions. Based on the Ramachandran plot results, all the predicted models, including HA, NA, PB2, and M2 protein, indicate a highly accurate homology model of each structure (Figure 2). Therefore, the Ramachandran plot and Verify-3D score suggest that the modeled proteins are stable. The score is closer to the expected high score (Table 1), showing that the predicted homology models of all protein structures are of good quality.

### 3.2. Results of the Binding Affinity of the Antiviral Compounds/Ligands with the Avian H5N1 Clade 2.2.4.4b HA Protein Using the Molecular Docking Approach

**Figure 3:**
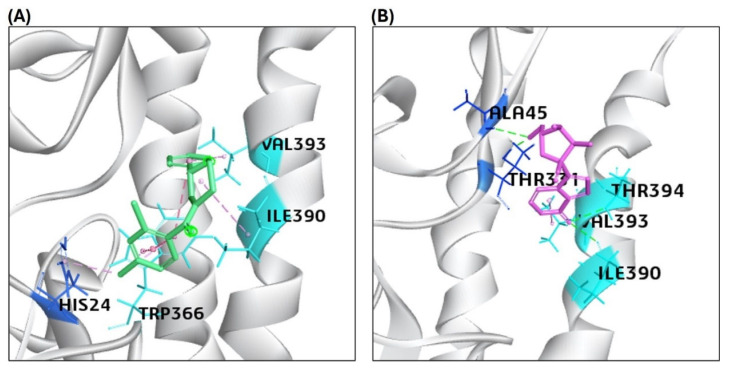
Representation of the molecular interaction of ligands in the binding sites of the structure of the HA protein. (**A**) F0045(S) (standard) interacted through a hydrogen bond with HIS24 of the HA1 chain, VAL 393, and ILE390 of the HA2 chain of the HA protein. (**B**) GS441524 interacted through hydrogen bonds with ALA45 and THR331 of the HA1 chain, VAL 393, and ILE390 of the HA2 chain of the HA protein.

Our molecular docking analysis revealed binding affinity and interactions of selected sofosbuvir and GS441524 ligands with interacting residues of HA, NA, PB2/CBD, and M2 protein. The compound IDs of all the ligands are listed in Table 2. The CDOCKER interaction energy scores suggested the binding affinity score (-CDOCK scores), as listed in Table 2. The GS445214 compound interacted with the active site residues of the same binding pocket as standard F0045(S) (Figure 3). The 2D interaction patterns of top hits of F0045(S) (standard) and GS441524 are illustrated in Figure S7.

**Figure 4:**
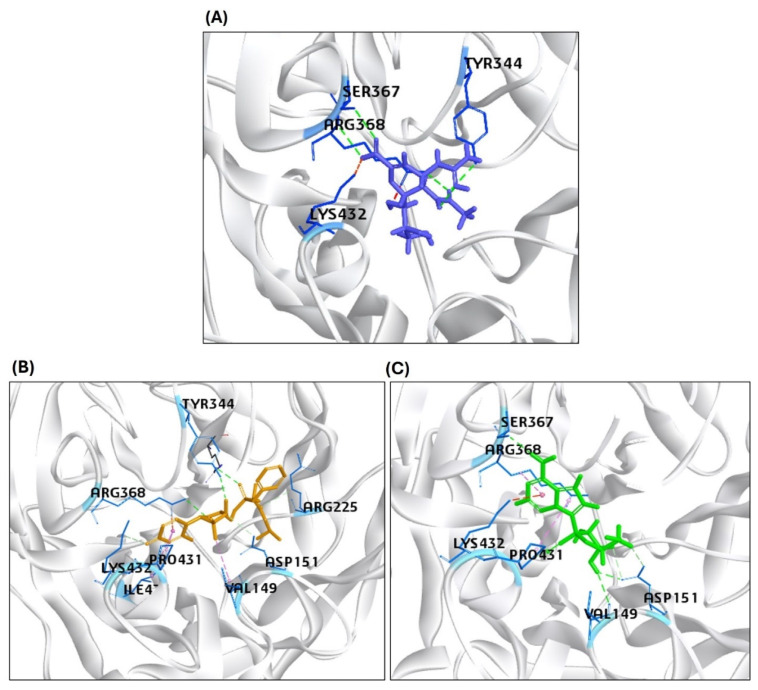
Representation of the molecular interaction of ligands in the binding sites of the structure of the NA protein. (**A**) Zanamivir (standard) interacted through hydrogen bonds with NA residues TYR344, ARG368, ASP151, and SER367. (**B**) Sofosbuvir established hydrogen bonds with NA residues TYR344, ARG368, TYR402, ARG225, LYS432, ASP151, and GLU278. (**C**) GS441524 interacted through hydrogen bonds with ARG368, VAL149, ASP151, SER367, PRO431 (three hydrogen bonds), and LYS432 residues of the NA protein.

The highest CDOCK scores (positive values) referred to the most favorable binding of the ligand to the protein. The HA protein was docked with GS445214 and standard compound F0045(S), with the -CDOCK scores of the HA-HA-GS441524 complex being 26.62, which is higher than the HA complex with a standard F0045(S), which is 22.92. The sofosbuvir was not docked in the same binding pocket of HA where the standard F0045(S) and GS441524 were docked. The standard ligand F0045(S) does not make any hydrogen bonds but makes hydrophobic interactions with interacting residues HIS24, TRP366, ILE390, and VAL393 of the HA protein. However, the best hit of GS441524 with HA established four hydrogen bonds with residues ALA45, THR331, ILE390, and THR394 in the active site of the target HA protein. The docking results of NA also showed that the -CDOCK score of Zanamivir (standard) with NA is 49.26. However, the -CDOCK scores of sofosbuvir and GS445214 with the NA are 53.43 and 55.30 (Table 2). The binding affinity score of the standard Zanamivir–NA complex is good, but low as compared to the -CDOCK score of sofosbuvir–NA and GS445214. Due to the interaction of Zanamivir and NA, seven hydrogen bonds were established, involving binding site residues TYR344, ARG368 (four hydrogen bonds), ASP151, and SER367. However, the top hit of ligand sofosbuvir established eight hydrogen bonds through NA protein interacting residues TYR344, ARG368, TYR402 (two hydrogen bonds), ARG225, LYS432, ASP151, and GLU278 in the active site. On the other hand, GS441524 established 10 hydrogen bonds with interacting residues ARG368 (four hydrogen bonds), VAL149, ASP151 (three hydrogen bonds), SER367, PRO431 (three hydrogen bonds), and LYS432. The GS445214 and sofosbuvir interacted with active site residues of the same binding pocket as Zanamivir (standard) (Figure 4). The 2D interaction patterns of top hits of Zanamivir (standard), sofosbuvir, and GS441524 are illustrated in Figure S8.

**Figure 5:**
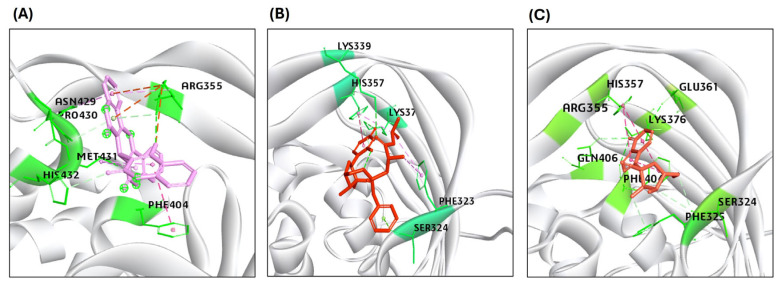
Results of the molecular interaction model of the tested compounds and the binding sites of the H5N1 clade 2.3.4.4b B2/CBD protein. (**A**) PB2-39 interacted through hydrogen bonds with only ARG355 and ASN429 residues of PB2/CBD. (**B**) Sofosbuvir interacted through hydrogen bonds with LYS339, LYS376, and HIS357. (**C**) GS441524 interacted through hydrogen bonds with ARG355, LYS376, PHE404, GLN406, GLU361, and SER324 residues in the binding site of the structure of the PB2/CBD protein.

Further, the docking of compound GS445214 and sofosbuvir with the PB2/CBD protein of H5N1 also showed promising results as compared to the standard compound PB2-39 for protein PB2-CBD. The binding affinity scores of sofosbuvir–PB2/CBD and GS441524 docked with PB2/CBD, as exhibited by the -CDOCK score, are 50.88 and 34.23. However, the -CDOCK score of the standard PB2-39 compound for PB2 is 54.55, slightly higher than the binding affinity score of PB2–sofosbuvir and PB2/CBD-GS441524 docking complex (Table 2). The ligand PB2-39 (standard) makes only two hydrogen bonds with interacting residues ARG355 and ASN429 of the PB2/CBD protein. The interaction of sofosbuvir with PB2/CBD established three hydrogen bonds with residues LYS339, LYS376, and HIS357 in the active site. However, the top hit of GS441524 with PB2/CBD suggested that six hydrogen bonds were established due to the interaction of GS441524 with residues ARG355, LYS376, PHE404, GLN406, GLU361, and SER324 in the active site of the PB2/CBD protein (Figure 5). The 2D interaction patterns of top hits of MGT and PB2-39 (standard), sofosbuvir, and GS441524 are illustrated in Figure S9. The presence of several hydrogen bonds, along with their favorable interaction score inside the binding cavity of the HA, NA, and PB2/CBD, predicts the validity of GS441524 as a potent inhibitor.

The transmembrane domain of the M2 protein is a tetrameric channel, the target of the anti-influenza drugs Amantadine and its methyl derivative Rimantadine. The M2 protein was docked with Rimantadine and Amantadine as the standard compounds. The CDOCK score of the M2–Rimantadine complex is 33.02, and that of the M2–Amantadine complex is 27.55 (Table 2).

The sofosbuvir showed a good binding affinity with M2 at the same binding pocket with the -CDOCKER score of 62.05, which is higher than that of the M2 complex with the Amantadine and Rimantadine compounds (Table 2).

**Figure 6:**
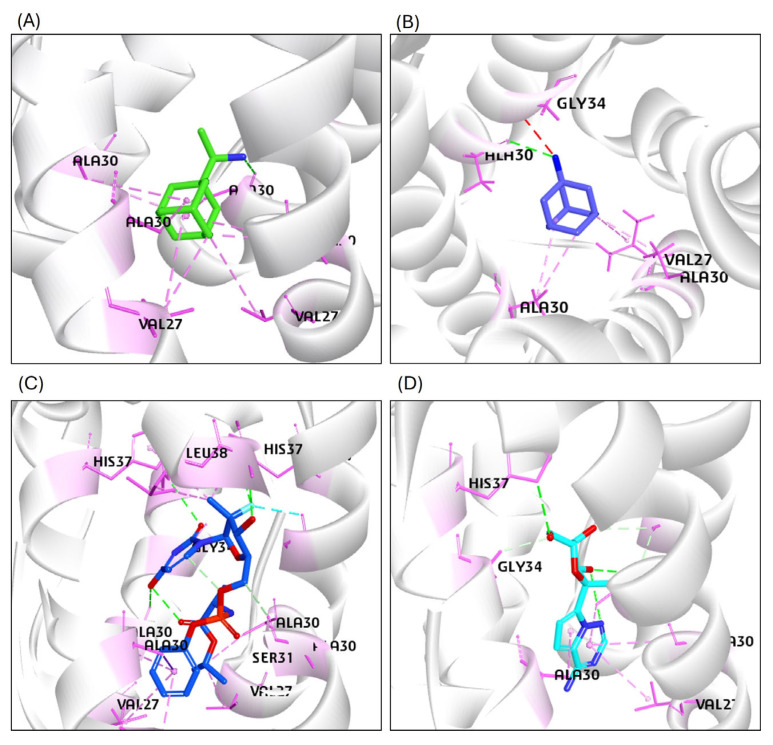
Results of the molecular interaction model of the tested compounds and the binding sites of the H5N1 clade 2.3.4.4b M2 tetrameric channel protein. (**A**) The Rimantadine (standard) interacted with the M2 protein residues of Chain-1 through hydrogen bonds: (AlA30; Chain 2: VAL27, ALA30; Chain 3: ALA30; Chain 4: VAL27, ALA30). (**B**) The Amantadine (standard) interacted with the M2 protein residues of Chain-2 through hydrogen bonds: (VAL27, ALA30); (Chain 3: ALA30); (Chain 4: ALA30, GLY34) amino acid residues of the M2 protein. (**C**) The sofosbuvir interacted through hydrogen bonds with M2-Chain 1: (VAL27, AlA30, GLY34, HIS37); Chain 2 through (ALA30, SER31, HIS37, LEU38); Chain 3 through (VAL27, ALA30, HIS37); and Chain 4 through (VAL27, ALA30, GLY34, HIS37) amino acid residues of the M2 protein. (**D**) The GS441524 compound interacted with hydrogen bonds with the M2-Chain 1: (AlA30, GLY34); Chain 2: (SER22, VAL27, ALA30); Chain 3: (ALA30, and HIS37) amino acid residues of the M2 channel protein.

The GS441524 compound docked with M2 exhibited a good binding affinity, with a CDOCK score of 42.59, which is comparatively higher than the binding affinity with the known antiviral drugs used as standards (Amantadine and Rimantadine). However, this binding affinity score is slightly lower than that of sofosbuvir with M2. The M2 interacting with standard ligands and the test ligands with established hydrogen bond and hydrophobic bond interactions are listed in Table 2. The top hits of the sofosbuvir and the GS441524 compounds with M2 created six and four hydrogen bonds, respectively (Figure 6). The 2D interaction patterns of the top hits of the Rimantadine, the Amantadine, sofosbuvir, and the GS441524 compounds are presented in Figure S10.

### 3.3. Results of the Calculation of the Ligands–H5N1 Clade 2.3.4.4b Proteins Binding Energy—(MM-GBSA)

The MM-GBSA calculations for the compounds GS441524 and sofosbuvir were performed using data from the top 10 docking hits, along with the respective standard compounds: F0045(s) for HA, PB2-39 for PB2/CBD, and Zanamivir for the NA protein.

Based on the interaction with the active site of the HA protein, GS441524 exhibited a binding energy of −61.43 kcal/mol, compared to −48.86 kcal/mol for the standard. In the case of NA protein interactions, the top docking hits of sofosbuvir and GS441524 showed binding free energies of −138.23 kcal/mol and −183.10 kcal/mol, respectively. These values were compared to the −201 kcal/mol binding energy of the top hit for the standard compound Zanamivir, indicating the complexes’ relative stability and supporting the docking results’ validity. However, the binding energy of GS441524 docked with the NA protein is considerable for further evaluation through molecular dynamics, as it is close to the binding energy of the standard docked complex of Zanamivir and NA.

The interaction of PB2/CBD with the standard compound PB2-39 resulted in a binding free energy of −236.79 kcal/mol, which is significantly more negative compared to the binding energies observed for PB2/CBD–sofosbuvir (−90.00 kcal/mol) and PB2/CBD-GS441524 (−199.00 kcal/mol). However, the binding energy of GS441524 docked with PB2/CBD is considerable for further evaluation through molecular dynamics, as it is close to the binding energy of the standard docked complex. These findings suggest greater complex stability for the standard compounds and support the reliability of the docking results (Table 2). For the M2 ion channel protein, the reference compounds Rimantadine and Amantadine showed binding energies of −112.23 kcal/mol and −104.46 kcal/mol, respectively. In contrast, the test compounds, sofosbuvir and GS441524, demonstrated more favorable (i.e., more negative) binding energies of −44.11 kcal/mol and −46.65 kcal/mol, indicating weaker interactions with the M2 protein than the standard Amantadine and Rimantadine compounds.

### 3.4. Molecular Dynamics Simulation Results for Avian H5N1 Clade 2.2.4.4b (HA, NA, PB2, and M2) Proteins and Some Selected Compounds/Drugs

Proteins play essential structural and functional roles in biological systems, including microbial pathogenesis, by mediating receptor-based internalization and replication. While molecular docking offers insights into the stability of protein–ligand complexes, it lacks the ability to capture their dynamic behavior over time. To address this limitation, molecular dynamics (MD) simulations were performed for 50 nanoseconds (ns) to evaluate the interactions of the GS441524 compound with the HA, NA, and PB2/CBD proteins of avian H5N1 clade 2.2.4.4b. Standard complexes—HA-F0045(S), NA–Zanamivir, and PB2/CBD-PB2-39—were also simulated under the same conditions for comparative analysis.

The MD simulations provided detailed insights into the molecular behavior of each complex by capturing time-dependent structural deviations and flexibility. Root Mean Square Deviation (RMSD) was used to assess conformational stability, while Root Mean Square Fluctuation (RMSF) and Radius of Gyration were calculated to evaluate residue-level mobility and compactness of the complexes, respectively. These parameters are critical for understanding the ligand-bound protein systems’ overall dynamics, structural stability, and flexibility. Additionally, hydrogen bond monitoring and hydrogen bond distance analyses were conducted to further explore each complex’s dynamic interactions and stability. The comparative trajectory analysis of GS441524-bound complexes (HA, NA, PB2/CBD) with their respective standards is presented and discussed in this section.

#### 3.4.1. Analysis of the RMSD Calculations Conferring Stability of Ligand–Protein Complexes

**Figure 7:**
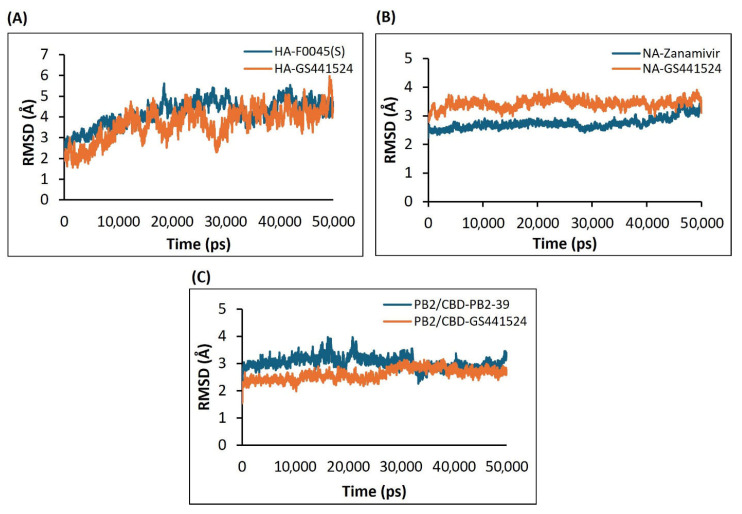
The RMSD plots based on the 50 ns molecular dynamics simulation trajectories for the H5N1 clade 2.3.4.4b proteins–ligands complexes: (**A**) HA in complex with the F0045(S) (standard) and GS441524; (**B**) NA in complex with the inhibitor Zanamivir (standard) and GS441524; (**C**) PB2/CBD in complex with the ligand PB2-39 (standard) and GS441524.

The RMSD plot analysis showed that all complexes, including the reference compounds, remained relatively stable when RMSD values were within the 3 Å threshold, as lower RMSD values typically indicate greater structural stability of protein–ligand complexes. For the HA-GS441524 complex, however, the RMSD values exhibited significant fluctuations compared to the HA-F0045(S) complex, suggesting lower dynamic stability of the GS441524-bound HA complex during the simulation (Figure 7A). The RMSD of the backbone atoms for HA-GS441524 and HA-F0045(S) ranged from approximately 1.8 Å to 5.5 Å. The average RMSD values were 3.2 Å for HA-GS441524 and 4.0 Å for HA-F0045(S), both exceeding the 3 Å stability threshold (Figure 6A). These results indicate that neither GS441524 nor the reference compound F0045(S) maintained consistent structural stability over the 50 ns simulation period.

The RMSD analysis of the neuraminidase (NA) complex with its standard inhibitor Zanamivir (NA–Zanamivir) revealed minimal structural fluctuations, with an average backbone RMSD of 3.13 Å—slightly exceeding the commonly accepted 3 Å stability threshold. In comparison, the NA–GS441524 complex showed a lower average RMSD of 2.5 Å (Figure 7B), suggesting enhanced structural stability. Thus, over the 50 ns simulation period, GS441524 exhibited superior dynamic stability when bound to the NA relative to the NA–Zanamivir complex.

RMSD analysis of the PB2/CBD complexes with PB2-39 and GS441524 indicated that the PB2/CBD–GS441524 complex exhibited the highest stability, maintaining RMSD values below 3 Å throughout the simulation. A slight increase to approximately 3 Å was noted after 30 ns; however, the RMSD remained steady between 2.7 Å and 3 Å from 30 to 50 ns without significant fluctuations (Figure 7C). In contrast, the reference complex PB2/CBD–F0045(S) displayed an average RMSD around 3 Å but underwent noticeable fluctuations between 17–21 ns and 32–35 ns. These findings suggest that GS441524 forms a more stable complex with PB2/CBD than the reference compound. Overall, the PB2 protein maintained a stable dynamic profile upon ligand binding, supporting the potential of GS441524 as an effective PB2 inhibitor.

#### 3.4.2. Analysis of the Stability and Mobility of the Interaction Between the H5N1 Clade 2.3.4.4b Proteins and the Selected Drugs/Compounds—RMSF

**Figure 8:**
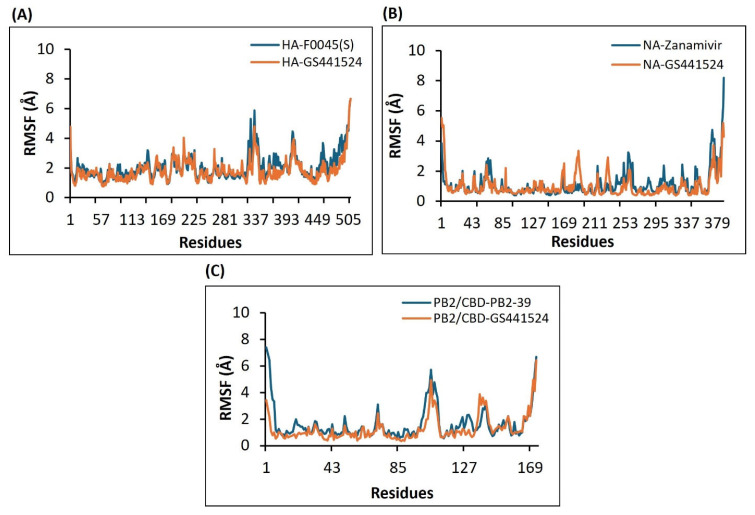
RMSF plots generated from the 50 ns MD simulation trajectories for the following protein–ligand complexes: (**A**) Model for HA in complex with F0045(S) (standard) or GS441524. (**B**) Model for NA in complex with Zanamivir (standard) or the GS441524 compound. (**C**) Model for the PB2/CBD complex with the PB2-39 (standard) or the GS441524 compound.

The RMSF (Root Mean Square Fluctuation) plots for the C-α atoms of all complexes were generated from the MD simulation trajectories. In both the HA–F0045(S) and the HA–GS441524 complexes, residues within the 337–393 region exhibited the highest flexibility. The HA–F0045(S) complex showed a peak fluctuation of approximately 6 Å, whereas the HA–GS441524 complex displayed slightly reduced fluctuations, peaking around 4.5 Å. Outside this region, most residues in both complexes fluctuated within a 0.5–2.5 Å range throughout the simulation. The overall RMSF profiles of the HA–GS441524 complex were comparable to those of the reference HA–F0045(S) complex, suggesting similar residue mobility and dynamic behavior (Figure 8A).

RMSF analysis of the NA–Zanamivir and NA–GS441524 complexes revealed that residues spanning positions 169 to 270 exhibited the most prominent fluctuations during the simulation. In this region, both complexes reached a maximum fluctuation of approximately 2.5 Å. Outside this range, most residues fluctuated between 0.5 and 1.5 Å over the 50 ns simulation period. The overall RMSF values for the NA–GS441524 complex were slightly lower but remained comparable to those of the NA–Zanamivir complex, suggesting similar residue flexibility and dynamic behavior in both complexes (Figure 8B).

The RMSF analysis of the PB2/CBD-PB2-39 and PB2/CBD-GS441524 complexes revealed that residues between positions 85 and 110 underwent the highest fluctuations during the simulation. The reference complex PB2/CBD-PB2-39 showed a peak fluctuation of approximately 6 Å, whereas the PB2/CBD-GS441524 complex exhibited a slightly lower peak of around 5 Å. Outside this region, residue fluctuations remained within 0.5–2.5 Å over the 50 ns trajectory. Notably, the PB2/CBD-GS441524 complex demonstrated lower overall RMSF values compared to the reference, suggesting reduced residue mobility and improved structural stability. Both complexes exhibited comparable fluctuation patterns within a 3 Å range around the same residue, indicating that the PB2 protein maintained its structural integrity upon ligand binding. These findings, reinforced by consistently low Radius of Gyration (Rg) values, highlight the compactness and stability of both the reference and test complexes (Figure 8C).

#### 3.4.3. Assessment of the Compactness and Structural Integrity of the H5N1 Clade 2.3.4.4b Proteins–Ligands Complex over Time Based on the Radius of Gyration (Rg) Using the Molecular Dynamics (MD) Simulations Approach

**Figure 9:**
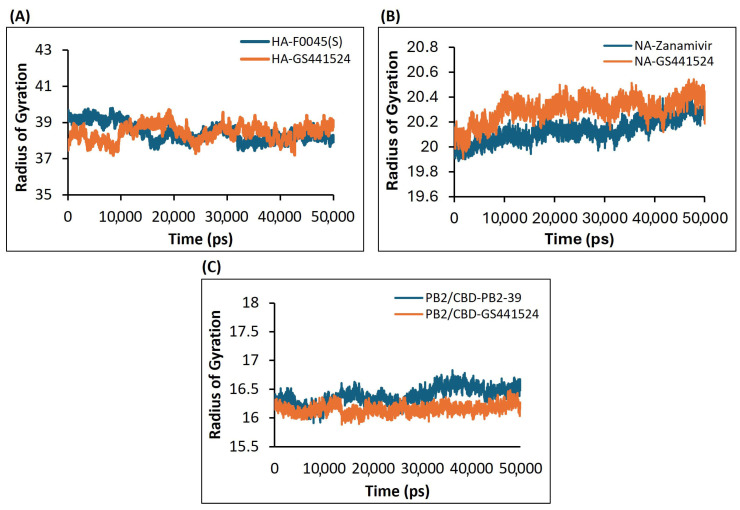
The Radius of Gyration (Rg) plots derived from the 50 ns molecular dynamics simulation trajectories for the following H5N1 clade 2.3.4.4b protein–ligand complexes: (**A**) HA-F0045(S) (standard) and HA-GS441524; (**B**) NA–Zanamivir (standard) and NA-GS441524; and (**C**) PB2/CBD-PB2-39 (standard) and PB2/CBD-GS441524.

The average Rg value for the HA-F0045(S) complex was approximately 38 Å, while the HA-GS441524 complex exhibited a slightly lower average Rg of about 37.5 Å (Figure 9A). These similar values indicate that HA-GS441524 maintains a comparable level of compactness to the reference complex. Throughout the simulation, the Rg of HA-GS441524 remained steady around or above 37.5 Å, with fewer fluctuations than the HA-F0045(S) complex, suggesting a relatively stable structural conformation. Although both complexes showed minor fluctuations, the HA-GS441524 complex appeared slightly less compact. Overall, all analyzed complexes demonstrated acceptable levels of compactness, with some conformational changes attributed to ligand binding.

In the case of the NA protein, the average Radius of Gyration (Rg) values for the NA–Zanamivir and NA-GS441524 complexes were 20.1 Å and 20.2 Å, respectively (Figure 9B). These closely aligned values suggest that both complexes preserved structural integrity throughout the simulation, exhibiting only minor fluctuations. Similarly, for the PB2/CBD protein, the PB2/CBD-PB2-39 complex had an average Rg of approximately 16.4 Å, whereas the PB2/CBD-GS441524 complex showed a slightly lower average Rg of 16.2 Å (Figure 9C). The lower Rg value and minimal variation in the PB2/CBD-GS441524 complex indicate a more compact and structurally stable configuration compared to the reference. Overall, the Rg analysis supports that all protein–ligand complexes presented in this study maintained satisfactory compactness during the 50 ns simulation, with no significant conformational changes induced by ligand binding.

#### 3.4.4. Assessment of H5N1 Clade 2.3.4.4b Proteins–Ligand Interactions Based on the Hydrogen Bonds Analysis of the Molecular Dynamics Simulations

The hydrogen bond (HB) formation in the complex during the simulation revealed the strength of the complex and its dynamic behavior, with each bond being formed within the specified time frame. To investigate the stability of the ligand interaction with the protein, the measurement of intermolecular hydrogen bond development and hydrogen bond distance between the ligand and interacting residues of proteins was evaluated.

**Figure 10:**
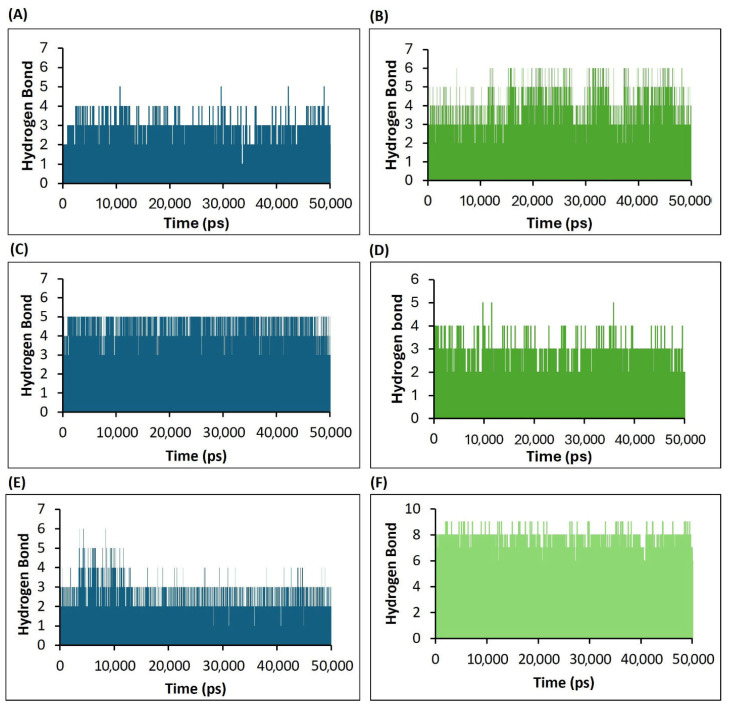
Analysis of the number of hydrogen bonds formed during interactions between H5N1 clade 2.3.4.4b proteins and ligands, as illustrated in the plots derived from the 50 ns molecular dynamics (MD) simulation trajectory data. (**A**) Results of the HA-F0045(S) (standard) complex. (**B**) Results of the HA-GS4415524 complex. (**C**) Results of the NA–Zanamivir (standard) complex. (**D**) Results of the NA-GS441524 complex. (**E**) Results of the PB2/CBD-PB2-39 (standard) complex. (**F**) Results of the PB2/CBD-GS441524 complex.

The hydrogen bond monitor of complex HA-F0045(S) suggested that around two hydrogen bonds formed throughout the simulation time frame. However, two other hydrogen bonds were also established in various frames during the simulation (Figure 10A). The complex HA-GS441524 formed four intermolecular hydrogen bonds throughout the simulation frames and two hydrogen bonds in various frames during the complete 50 ns simulation time (Figure 10B).

In the case of NA protein hydrogen bond monitoring, the complex NA–Zanamivir (standard) maintained four hydrogen bonds throughout the entire simulation time (Figure 10C). However, the complex NA-GS441524 formed three stable hydrogen bonds (Figure 10D).

The hydrogen bond analysis of the PB2/CBD complex with the standard ligand PB2-39 (PB2/CBD-PB2-39) revealed the formation of two stable hydrogen bonds throughout the 50 ns simulation (Figure 10E), with two additional hydrogen bonds forming intermittently. In contrast, the PB2/CBD-GS441524 complex consistently maintained five stable hydrogen bonds, along with two more that appeared intermittently during the simulation (Figure 10F). These results indicate that the PB2/CBD-GS441524 complex exhibited greater stability compared to the reference PB2/CBD-PB2-39 complex.

#### 3.4.5. Results of the Estimation of the Hydrogen Bond Distances as a Measure of the Complex Stability Between the H5N1 Clade 2.3.4.4b with the Selected Ligands

To evaluate the stability of hydrogen bonds, changes in the distances between the compounds and key amino acid residues involved in hydrogen bonding were analyzed throughout the MD simulations. This was performed by monitoring the variation in distance between the Cα atoms of the binding pocket and the ligands. For stable hydrogen bonds, the average distance should ideally remain below 3 Å.

**Figure 11:**
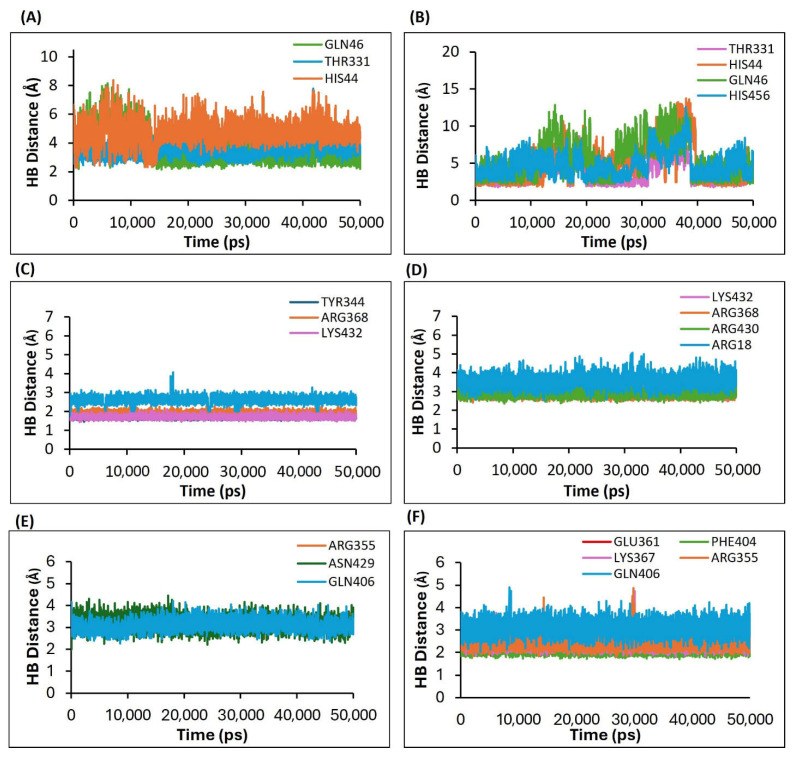
Plots depicting the variation in hydrogen bond distances between ligands and interacting protein residues, based on the 50 ns MD simulation trajectories for the following complexes. (**A**) HA-F0045(S) (standard), (**B**) HA-GS4415524, (**C**) NA–Zanamivir (standard), (**D**) NA-GS441524, (**E**) PB2/CBD-PB2-39 (standard), and (**F**) PB2/CBD-GS441524.

In the HA-F0045(S) complex, the standard ligand F0045(S) formed three hydrogen bonds with residues GLN46, THR331, and HIS44 of the HA protein. The average bond distances were approximately 3.0 Å, 3.1 Å, and 5.0 Å, respectively (Figure 11A). However, these interactions showed fluctuations ranging from 2.2 Å to 7.0 Å over the course of the simulation, indicating relatively unstable bonding, particularly in the case of HIS44.

In contrast, the HA-GS441524 complex displayed interactions between GS441524 and four HA residues: THR331, HIS44, GLN46, and HIS465. The average hydrogen bond distances were about 3.2 Å, 3.3 Å, 4.1 Å, and 4.2 Å, respectively, with fluctuations ranging from 2.9 Å to 12.0 Å (Figure 11B). These larger variations further suggest that the interactions between GS441524 and the HA protein were also relatively unstable during the simulation.

For the NA–Zanamivir (standard) complex, three hydrogen bonds were observed between Zanamivir and NA protein residues TYR344, ARG368, and LYS432. The average hydrogen bond distances were 2.0 Å, 2.5 Å, and 2.9 Å, respectively, indicating stable interactions between Zanamivir and the NA protein (Figure 11C).

In comparison, the NA–GS441524 complex exhibited four hydrogen bonds involving residues LYS432, ARG368, ARG430, and ARG18 of the NA protein. The average bond distances were 2.3 Å, 3.0 Å, 2.2 Å, and 3.5 Å, respectively, most of which fall within the optimal hydrogen bonding range of 2.5–3.0 Å. Notably, GS441524 formed an additional hydrogen bond compared to Zanamivir, suggesting a broader interaction profile. These interactions indicate that GS441524 maintains a comparable level of binding stability and may offer favorable binding characteristics as a potential NA inhibitor (Figure 11D).

For the PB2/CBD-PB2-39 (standard) complex, PB2-39 established three hydrogen bonds with ARG355, ASN429, and GLN406 residues of PB2/CBD, with average bond distances of 3.0 Å, 3.1 Å, and 3.3 Å, respectively (Figure 11E).

In contrast, for the PB2/CBD-GS441524 complex, GS441524 formed five hydrogen bonds with PB2/CBD residues including GLU361, PHE404, LYS367, ARG355, and GLN406. The average hydrogen bond distances were 2.0 Å, 2.2 Å, 2.3 Å, 2.5 Å, and 3.0 Å, respectively, indicating more stable interactions than those seen in the PB2/CBD-PB2-39 complex (Figure 11F).

## 4. Discussion

This study utilized an integrated computational strategy—including homology modeling, molecular docking, molecular dynamics (MD) simulations, and MM-GBSA binding free energy calculations—to evaluate the structural stability and inhibitory potential of GS441524 and sofosbuvir against key viral proteins of the currently circulating H5N1 clade 2.3.4.4b, namely hemagglutinin (HA), neuraminidase (NA), polymerase basic protein 2 C-terminal domain (PB2/CBD), and matrix protein 2 (M2). These proteins are critical for viral entry, replication, and host adaptation, making them promising therapeutic targets for combating avian influenza [36,37,38,39].

The high-quality homology models of HA, NA, and PB2/CBD were validated using multiple structural assessment parameters, including favorable DOPE scores, low PDF total energy values, and Ramachandran plot analyses. Most amino acid residues were located within favored regions of the Ramachandran plots, confirming appropriate backbone dihedral angles and overall model stability. Additionally, the Verify-3D scores for all models exceeded the minimum acceptable thresholds and approached or surpassed expected high-quality benchmarks, further supporting the structural reliability of the predicted protein models [40]. These findings confirmed the reliability of the models for use in subsequent docking and simulation analyses.

Molecular docking results revealed that GS441524 and sofosbuvir exhibit strong binding affinities toward the HA, NA, and PB2/CBD proteins, often matching or surpassing those of established reference ligands (Table 2). Notably, GS441524 showed higher CDOCKER interaction energy scores than F0045(S) when docked with HA and formed multiple hydrogen bonds with critical residues, such as ILE390 and THR394, which play key roles in the viral fusion process [41]. Similarly, both sofosbuvir and GS441524 exhibited strong interactions with the NA protein, forming up to 10 hydrogen bonds with key catalytic residues such as ARG368 and ASP151, which are essential for neuraminidase enzymatic function [42].

The PB2-39 is among the identified PB2/CBD inhibitors and has been recognized as a potent agent against the replication of various influenza A virus subtypes. When used in combination with the viral release inhibitor Zanamivir, PB2-39 demonstrated a synergistic antiviral effect, enhancing the overall efficacy of the treatment [38].

In the PB2/CBD docking analysis, GS441524 showed extensive interactions with catalytically and structurally significant residues, such as ARG355 and PHE404, forming multiple hydrogen bonds. Its binding energy was comparable to, or slightly lower than, that of the standard compound PB2-39. MM-GBSA calculations supported these findings, revealing favorable binding energies for GS441524 with HA (−61.43 kcal/mol), NA (−83.10 kcal/mol), and PB2/CBD (−99.08 kcal/mol) (Table 2). Notably, the binding energy of GS441524 with NA exceeded that of Zanamivir, a clinically approved neuraminidase inhibitor [43]. These results highlight GS441524′s potential as a viable anti-influenza agent. Although its binding energies with PB2/CBD were slightly less favorable compared to MGT or PB2-39, the overall interaction profile and energy scores reinforce its promise as a strong candidate for inhibiting influenza virus replication.

The M2 tetrameric channel protein of the influenza A virus serves as a crucial membrane-bound proton channel that acidifies virions enclosed within endosomes, thereby mediating the viral uncoating process and enabling the release of viral RNA into the host cell cytoplasm [44]. In our molecular docking analysis, the binding affinities of standard antiviral agents (Amantadine and Rimantadine) and test compounds (sofosbuvir and GS441524) were assessed against the M2 transmembrane domain of influenza A virus. Rimantadine and Amantadine exhibited moderate binding affinities, with CDOCKER interaction scores of 33.02 and 27.55, respectively, and corresponding binding energies of −112.23 kcal/mol and −104.46 kcal/mol. However, known mutations within the M2 membrane-spanning domain that confer resistance to Amantadine have been shown to generate ion channel activity that is no longer inhibited by the drug [45].

The binding sites for Amantadine and Rimantadine within the M2 ion channel are located near the histidine gate (His37) and pore-lining residues (Leu26, Ala30, and Ser31), where these inhibitors function as channel blockers. Binding interaction analysis showed that both standard and test compounds formed key hydrogen bonds and hydrophobic interactions with conserved residues of the H5N1 clade 2.3.4.4b M2 channel (Table 2). Notably, the test compound sofosbuvir demonstrated a substantially higher CDOCKER score (62.05) and more favorable binding energy (−44.11 kcal/mol) than the standard inhibitors, suggesting a stronger and more stable interaction with the M2 channel. Likewise, GS441524—the parent nucleoside of Remdesivir—resulted in a solid CDOCKER score of 42.59 and the most negative binding energy (−44.65 kcal/mol) among all tested compounds. This indicates a lower binding affinity and a robust interaction with the M2 protein, particularly through engagement with key residues such as Val27, Ala30, and His37. These findings, closely aligned with known interaction patterns of standard drugs, support the potential of GS441524 as a promising M2 channel inhibitor and affirm the reliability of the docking analysis [46,47,48].

Based on the binding free energy calculations (Table 2), HA, NA, and PB2/CBD protein targets were selected for further evaluation using molecular dynamics simulations, while the M2 protein was excluded. GS441524 exhibited a notably higher binding energy (−61.43 kcal/mol) than the standard F0045(S) (−48.86 kcal/mol) for HA, indicating favorable and stable interactions. For the NA protein, although Zanamivir showed the most negative binding energy (−201 kcal/mol), GS441524 (−183.10 kcal/mol) and sofosbuvir (−138.23 kcal/mol) also demonstrated substantial binding affinity, supporting their continued analysis. In the case of PB2/CBD, GS441524 showed a binding energy of −199.00 kcal/mol, which is comparable to the standard PB2-39 (−236.79 kcal/mol), justifying its inclusion in the MD simulation. Conversely, sofosbuvir and GS441524 exhibited weaker interactions with the M2 protein, with binding energies of −44.11 kcal/mol and −46.65 kcal/mol, respectively—significantly less favorable than those of the standard inhibitors Rimantadine and Amantadine. Due to these comparatively weaker affinities, the M2 protein was omitted from further simulation studies.

The molecular dynamics simulations conducted over a 50 ns period revealed varying levels of stability across the protein–ligand complexes. In the case of HA, both GS441524 and the reference ligand F0045(S) showed elevated RMSD values exceeding 3 Å, indicating moderate conformational fluctuations and reduced stability. In contrast, the GS441524 complexes with NA and PB2/CBD displayed consistent RMSD values at or below 3 Å throughout the simulation, reflecting strong dynamic stability and limited structural deviations [49,50]. The RMSF analysis revealed minimal fluctuations at the residue level across all complexes, with PB2/CBD-GS441524 exhibiting slightly greater residue stability compared to the reference PB2-39, indicating tighter binding and decreased flexibility. Additionally, the Radius of Gyration (Rg) measurements confirmed the overall structural compactness of the ligand–protein complexes. Notably, PB2/CBD-GS441524 displayed a more compact and stable conformation than the standard reference, further supporting its enhanced binding affinity and stability [51,52].

The hydrogen bonding analysis of GS441524 with NA demonstrated favorable interaction patterns, although these were slightly less stable compared to those observed with PB2/CBD. GS441524 formed more hydrogen bonds with HA than the reference ligand F0045(S), but the dynamic stability of this complex was comparatively weaker, indicating that further optimization may be needed to improve binding efficacy with both HA and NA. Conversely, hydrogen bond analysis strongly supported the stability of GS441524′s interaction with PB2/CBD. In the PB2/CBD-GS441524 complex, up to five persistent hydrogen bonds were maintained, with bond lengths consistently below 3 Å, reflecting stable and specific binding. This suggests GS441524 is a promising inhibitor of PB2/CBD, potentially outperforming the standard inhibitor PB2-39. These findings are especially important given the critical role of the PB2 protein in viral polymerase function and host adaptation, where its inhibition could significantly disrupt viral replication [53,54].

The molecular docking and molecular dynamics simulation results from our study are consistent with previous studies that have demonstrated the broad-spectrum antiviral efficacy of GS441524 against various RNA viruses, including SARS-CoV-2 and feline infectious peritonitis virus (FIPV) [27,55,56,57]. Its ability to target NA and PB2/CBD with good binding affinity, a greater number of hydrogen bonds and less hydrogen bond distance measurements as compared to the standards, during MD simulation, indicates that GS441524 holds promise as a novel therapeutic candidate against avian influenza strains like H5N1, which remain significant zoonotic and pandemic threats. In summary, this study offers valuable insights into the potential of GS441524 for drug repurposing and highlights the effectiveness of computational methods in accelerating the discovery of broad-spectrum antiviral agents against emerging and re-emerging viral pathogens.

## 5. Conclusions

This comprehensive in silico study provides strong evidence that GS441524, the active metabolite of Remdesivir, is a promising multi-target antiviral agent against H5N1 avian influenza virus clade 2.3.4.4v. Using structural modeling, molecular docking, MM-GBSA binding energy calculations, and 50 ns molecular dynamics simulations, GS441524 demonstrated stable and high-affinity binding to four key viral proteins: hemagglutinin (HA), neuraminidase (NA), Matrix-2 (M2), and the cap-binding domain of PB2 (PB2/CBD). Among these, GS441524 showed especially notable dynamic stability and binding strength with NA and PB2/CBD, supported by favorable -CDOCKER scores, robust hydrogen bonding with less hydrogen bond distance, consistent RMSD/RMSF values, lower Radius of Gyration, and shorter hydrogen bond distances. These results suggest GS441524′s potential dual inhibition of viral entry and replication. Consistent with prior reports of its broad-spectrum antiviral activity against multiple RNA viruses, GS441524′s targeting of conserved influenza protein domains positions it as a viable candidate for repurposing against H5N1, a significant zoonotic and pandemic threat. However, in silico findings require experimental validation. Future work should include in vitro antiviral testing, in vivo efficacy and toxicity studies, pharmacokinetics, and exploration of resistance mechanisms. Evaluating combination therapies with existing antivirals like PB2-39, Zanamivir, Oseltamivir, or Baloxavir could further advance GS441524′s clinical development.

## Figures and Tables

**Table 1:** List of the predicted homology models of the major proteins of the H5N1 clade 2.3.4.4b and their verification parameters.

N	Predicted Homology Model	PDF Total Energy	DOPE Score	Verify Score	Verify Expected High Score	Expected Low Score
1	HA	26,755	−54,478	205	232	104
2	NA	18,118	−43,552	180	176	79
3	PB2/CBD	1926	−79,787	277	333	154
4	M2	5702	−10,588	31	45	20

**Table 2:** Summary of the binding affinity scores, the binding free energies, and the molecular interactions between the avian H5N1 clade 2.2.4.4b (HA, NA, PB2/CBD, and M2) proteins and their respective ligands.

S. No	Protein	Ligands/ Compound-ID	MM-GBSA Binding Energy (kcal/mol)	-CDOCK Score (Binding Affinity Score)	Interacting Residues
1	HA	* F0045(S) (CID: 25281297)	−48.864	22.9215	HIS24, TRP366, ILE390, VAL393
2	HA	GS441524 (CID: 44468216)	−61.432	26.672	ALA45, THR331, ILE390, VAL393, THR394
3	PB2/CBD	* PB2-39 (CID: 54694510)	−236.799	54.5527	PHE404, ASN429, PRO430, HIS432, ARG355
4	PB2/CBD	Sofosbuvir (CID: 45375808)	−110.053	50.8859	PHE323, SER324, LYS339, HIS357, LYS367
5	PB2/CBD	GS441524 (CID: 44468216)	−199.079	34.2346	SER324, ARG355, HIS357, PHE404, GLN406, GLU361, LYS376
6	NA	* Zanamivir (CID: 60855)	−201	49.26	ASP151, TYR344, SER367, ARG368, LYS432
7	NA	GS441524 (CID: 44468216)	−183.10	55.30	VAL149, ASP151, SER367, ARG368, PRO431, LYS432
8	NA	Sofosbuvir (CID: 45375808)	−138.23	53.43	VAL149, ASP151, ARG225, GLU278, TYR344, ARG368, TYR402, ILE427, PRO431, LYS432
9	M2	Rimantadine (CID: 5071)	−112.23	33.02	Chain 1: ALA30; Chain 2: VAL27, ALA30 Chain 3: ALA30; Chain 4: VAL27, ALA30
10	M2	Amantadine (CID: 5071)	−104.46	27.55	Chain 2: VAL27, ALA30; Chain 3: ALA30; Chain 4: ALA30, GLY34
11	M2	Sofosbuvir (CID: 45375808)	−44.11	62.05	Chain 1: VAL27, AlA30, GLY34, HIS37; Chain 2: ALA30, SER31, HIS37, LEU38 Chain 3: VAL27, ALA30, HIS37 Chain 4: VAL27, ALA30, GLY34, HIS37
12	M2	GS441524 (CID: 44468216)	−46.65	42.59	Chain 1: AlA30, GLY34. Chain 2: SER22, VAL27, ALA30 Chain 3: ALA30, HIS37

* Represents the standard ligands for HA, NA, and PB2. -CDOCK score refers to the CDOCKER interaction energy.

## Data Availability

The raw data supporting the conclusions of this article will be made available by the authors upon request.

## Supplementary Materials

The following supporting information can be downloaded at https://www.mdpi.com/article/10.3390/v17070972/s1; Figure S1: Superimposition of the predicted homology models of (A) HA, (B) NA, (C) PB2/CBD, and (D) M2 proteins with their respective template structures used for model generation. Figure S2: The multiple sequence alignment result of BLAST Search for the HA query sequence to find identical and similar templates. The query sequence of HA (Top) is aligned with templates sequences to predict the 3D protein structure of HA. The PDB IDs of the templates sequences are given in below query sequence HA. Figure S3: The multiple sequence alignment result of BLAST Search for the NA query sequence to find identical and similar templates. The query sequence of NA (Top) is aligned with templates sequences to predict the 3D protein structure of NA. The PDB IDs of the templates sequences are given below query sequence NA. Figure S4: The multiple sequence alignment result of BLAST Search for the PB2 sequence carrying CBD sequence to find identical and similar templates. The query sequence of PB2 (Top) is aligned with templates sequences to predict the 3D protein structure of PB2. The PDB IDs of the templates sequences are given below query sequence PB2. Figure S5: The multiple sequence alignment result of BLAST Search for the Matrix 2 (M2) protein channel sequence to find identical and similar templates. The query sequence of M2 named as matrix in the figure is aligned with template (2KQT) sequences to predict the 3D protein structure of PB2. The PDB IDs of the templates sequences are given above query sequence PB2. Figure S6: 3D image of the predicted homology models of proteins (A) HA, (B) NA, (C) PB2/CBD and (D) M2. Figure S7: The 2D docked conformation of (A) F0045(S) (B) GS441524 in binding site of HA protein of H5N1. Figure S8: The 2D docked conformation of (A) Zanamivir (B) Sofosbuvir and (C) GS441524 in active site of NA protein of H5N1. Figure S9: The 2D docked conformation of (A) PB2-39 (B) Sofosbuvir and (C) GS441524 in active site of PB2/CBD protein of H5N1. Figure S10: The 2D docked conformation of (A) Rimantadine (B) Amantadine (C) Sofosbuvir and (D) GS441524 in active site of M2 channel protein of H5N1.
