# Supplementary material for: Repurposing of Some Nucleoside Analogs Targeting Some Key Proteins of the Avian H5N1 Clade 2.3.4.4b to Combat the Circulating HPAI in Birds: An In Silico Approach
Source: Viruses. 2025 Jul 10;17(7):972. doi: 10.3390/v17070972 (PMC12299010; doi:10.3390/v17070972)
Supplement: Supplementary file 1 [file viruses-17-00972-s001.zip › viruses-3706253-supplementary.pdf]

viruses

MDPI

Supplementary Material

Repurposing of Antiviral Inhibitors Targeting Key Proteins of Avian H5N1 Clade 2.3.4.4b via In Silico Approaches

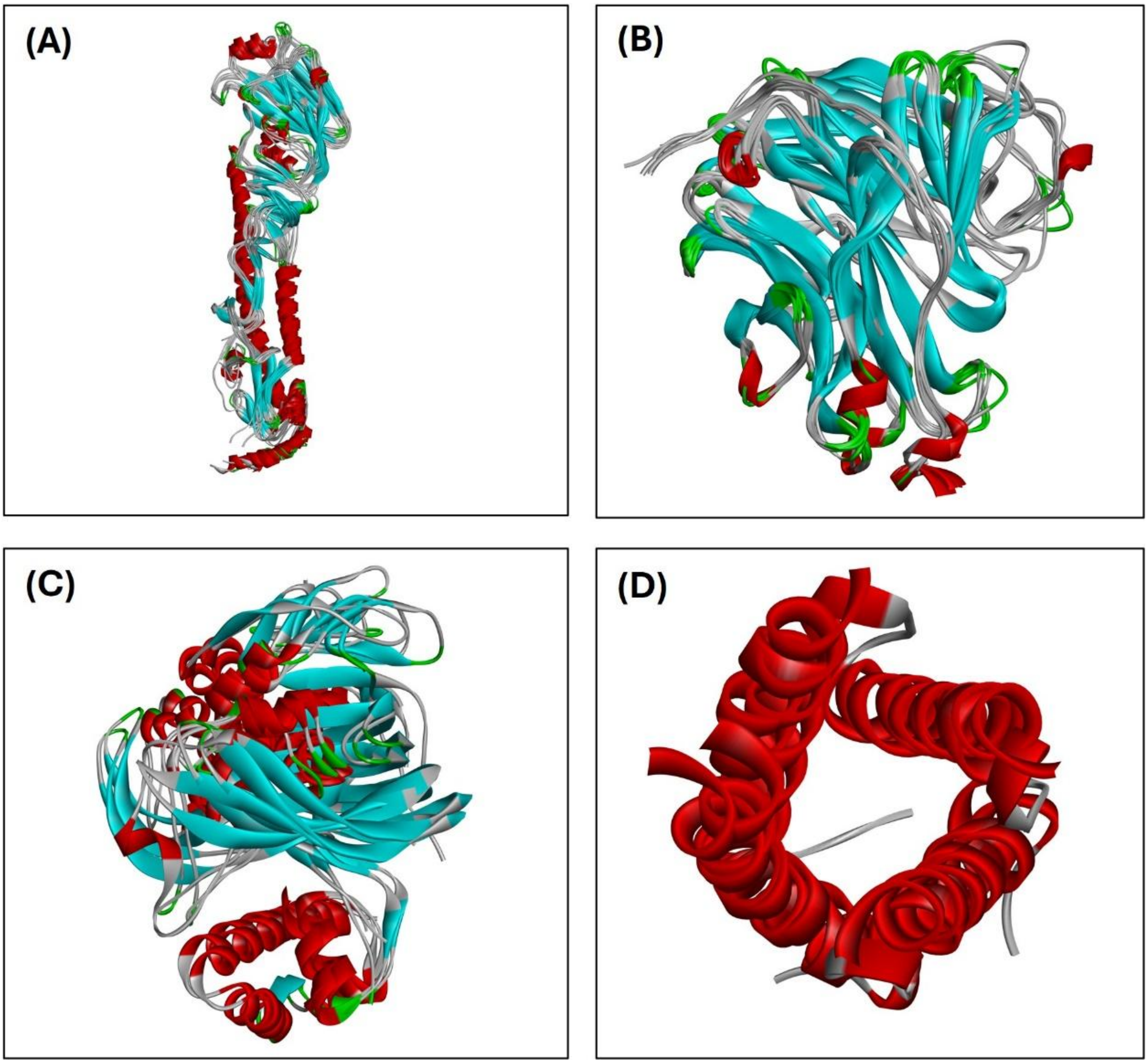

Figure S1. Superimposition of the predicted homology models of **(A)** HA, **(B)** NA, **(C)** PB2/CBD, and **(D)** M2 proteins with their respective template structures used for model generation.

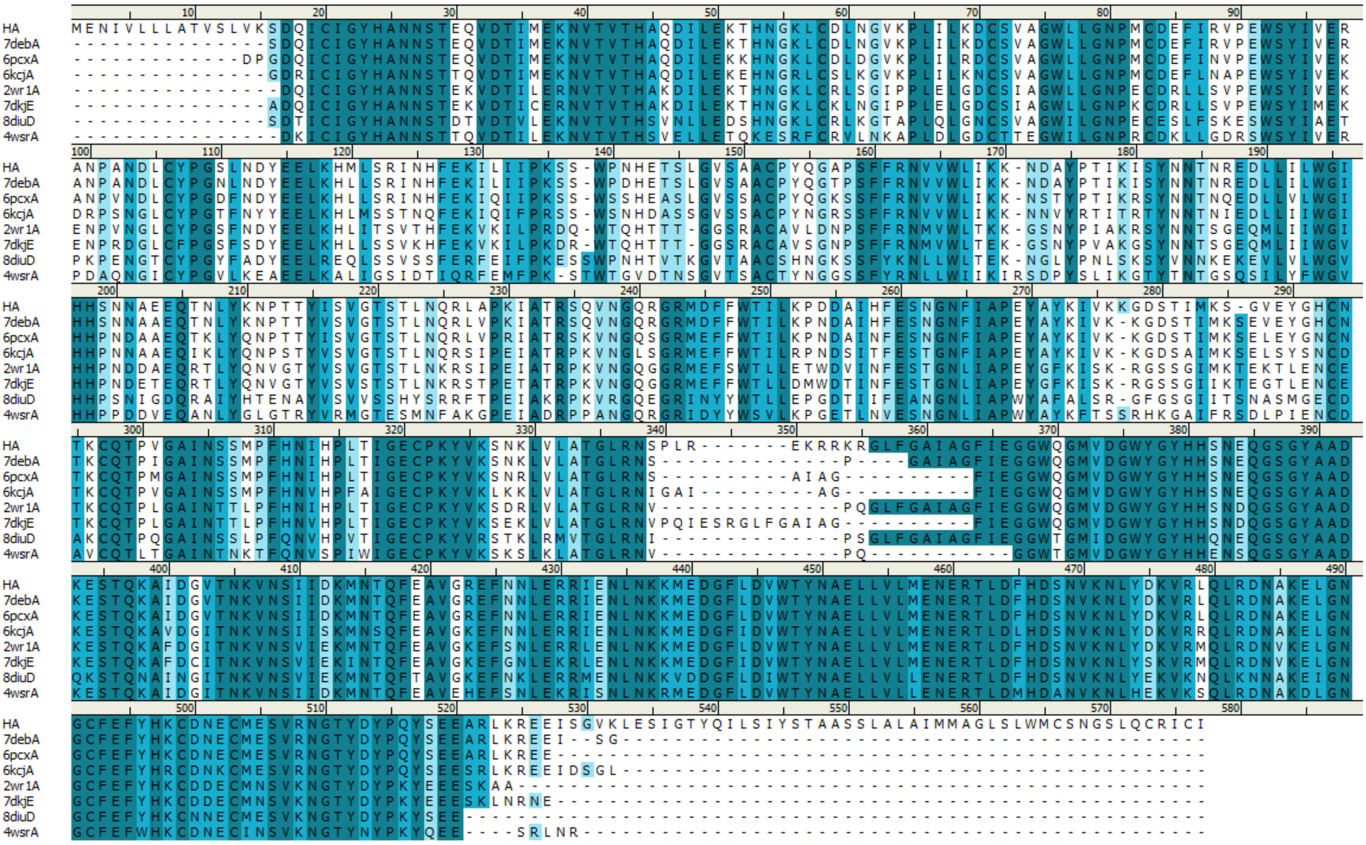

Figure S2. The multiple sequence alignment result of BLAST Search for the HA query sequence to find identical and similar templates. The query sequence of HA (Top) is aligned with templates sequences to predict the 3D protein structure of HA. The PDB IDs of the templates sequences are given in below query sequence HA.

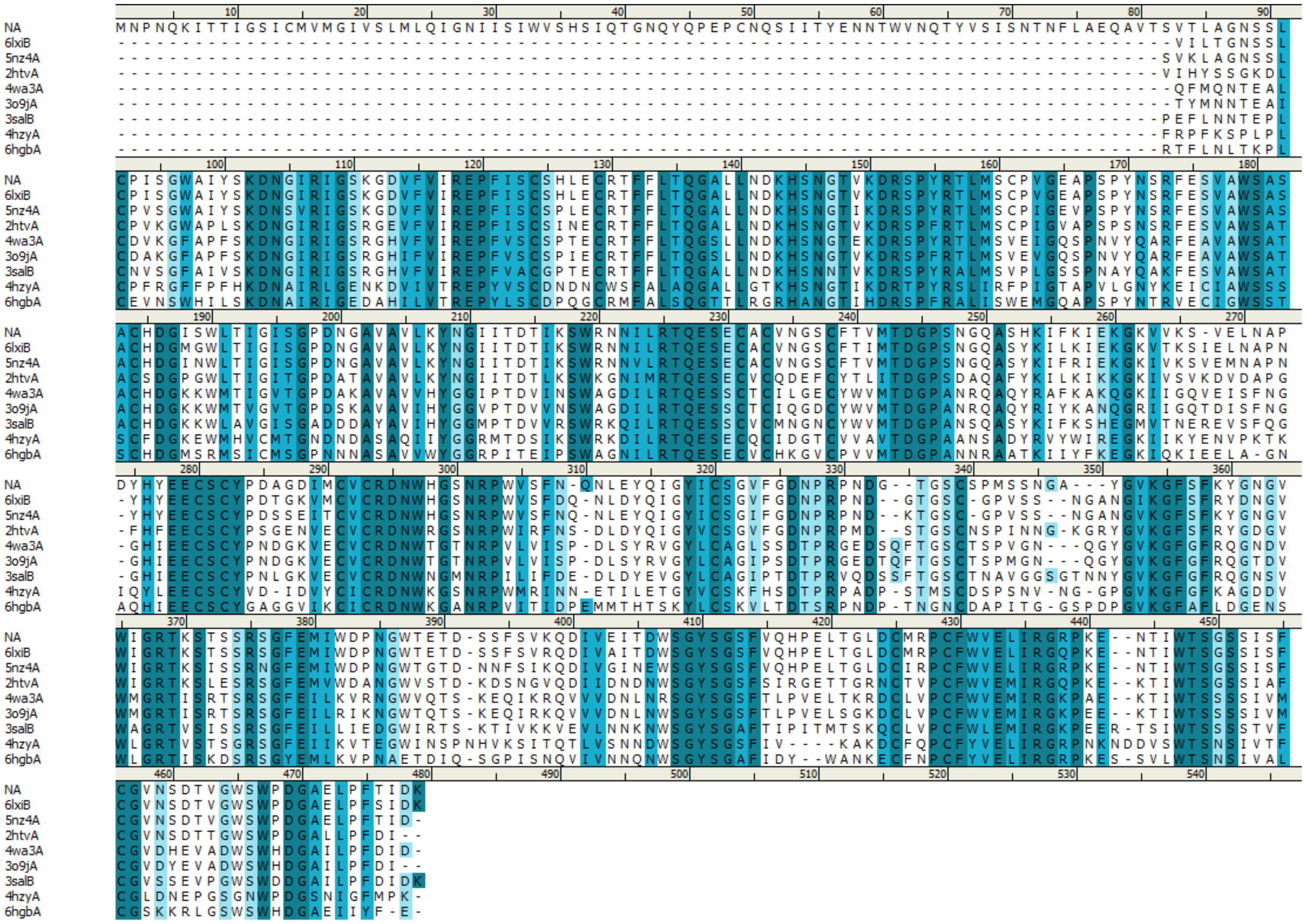

Figure S3. The multiple sequence alignment result of BLAST Search for the NA query sequence to find identical and similar templates. The query sequence of NA (Top) is aligned with templates sequences to predict the 3D protein structure of NA. The PDB IDs of the templates sequences are given below query sequence NA.

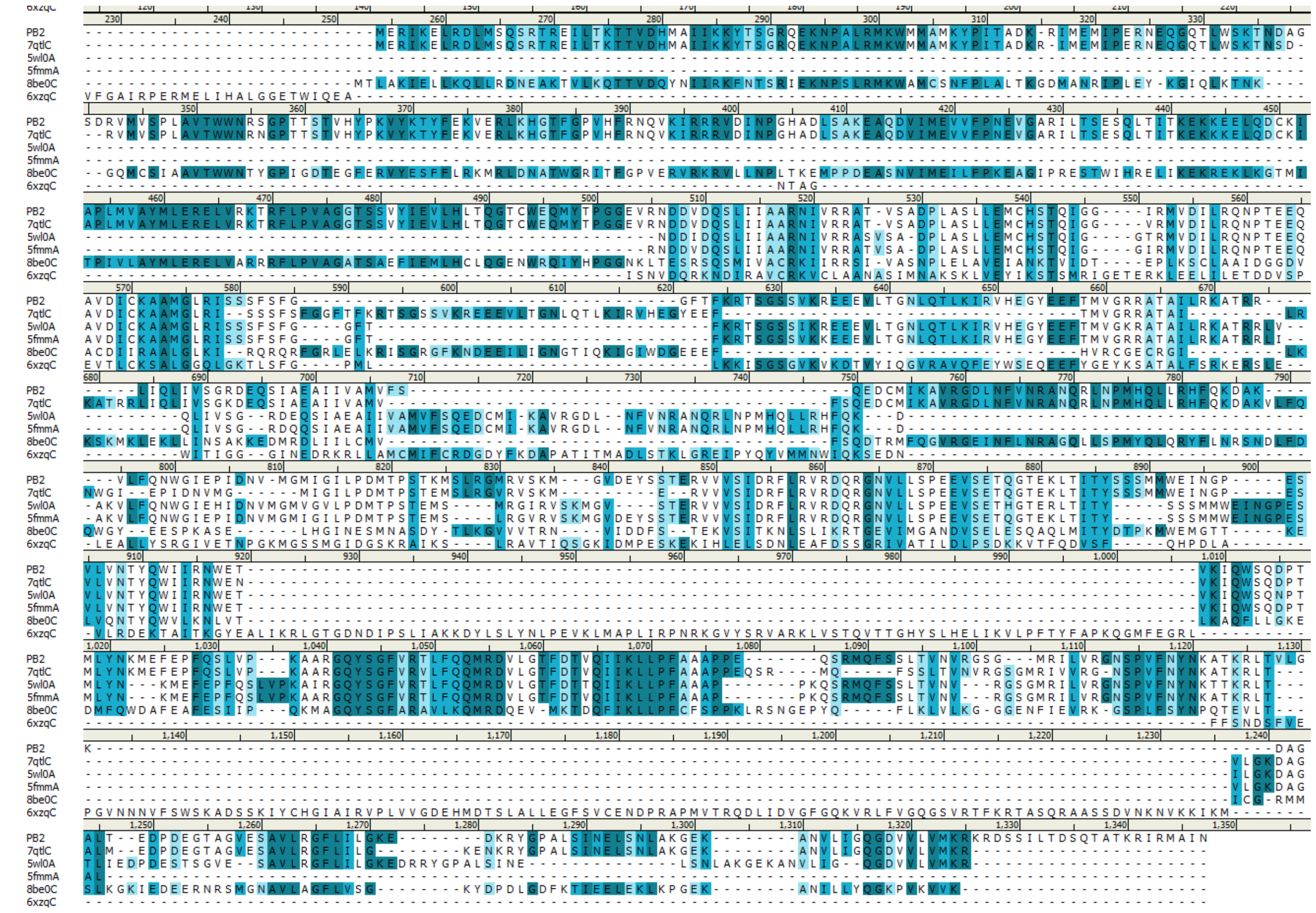

Figure S4. The multiple sequence alignment result of BLAST Search for the PB2 sequence carrying CBD sequence to find identical and similar templates. The query sequence of PB2 (Top) is aligned with templates sequences to predict the 3D protein structure of PB2. The PDB IDs of the templates sequences are given below query sequence PB2.

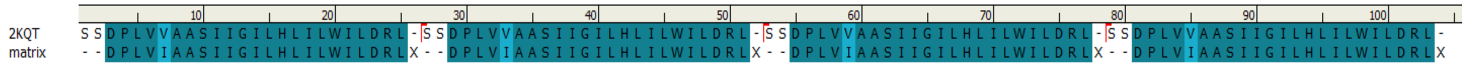

Figure S5. The multiple sequence alignment result of BLAST Search for the Matrix 2 (M2) protein channel sequence to find identical and similar templates. The query sequence of M2 named as matrix in the figure is aligned with template (2KQT) sequences to predict the 3D protein structure of PB2. The PDB IDs of the templates sequences are given above query sequence PB2.

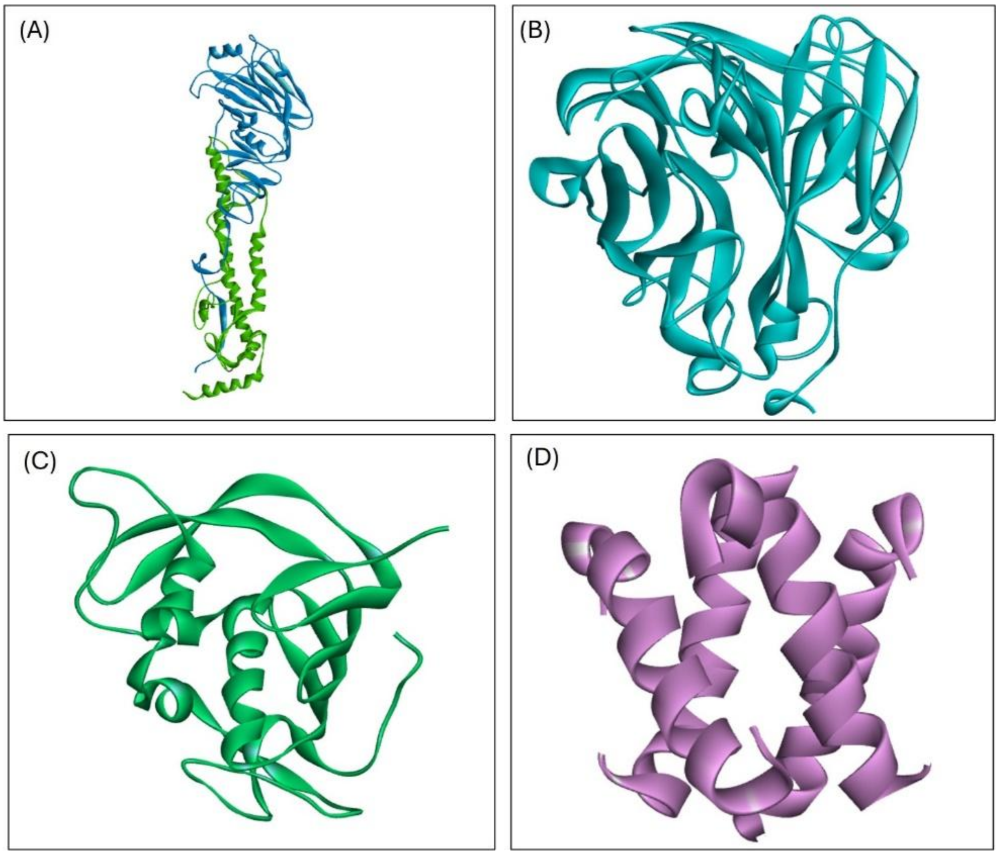

Figure S6. 3D image of the predicted homology models of proteins (A) HA, (B) NA, (C) PB2/CBD and (D) M2.

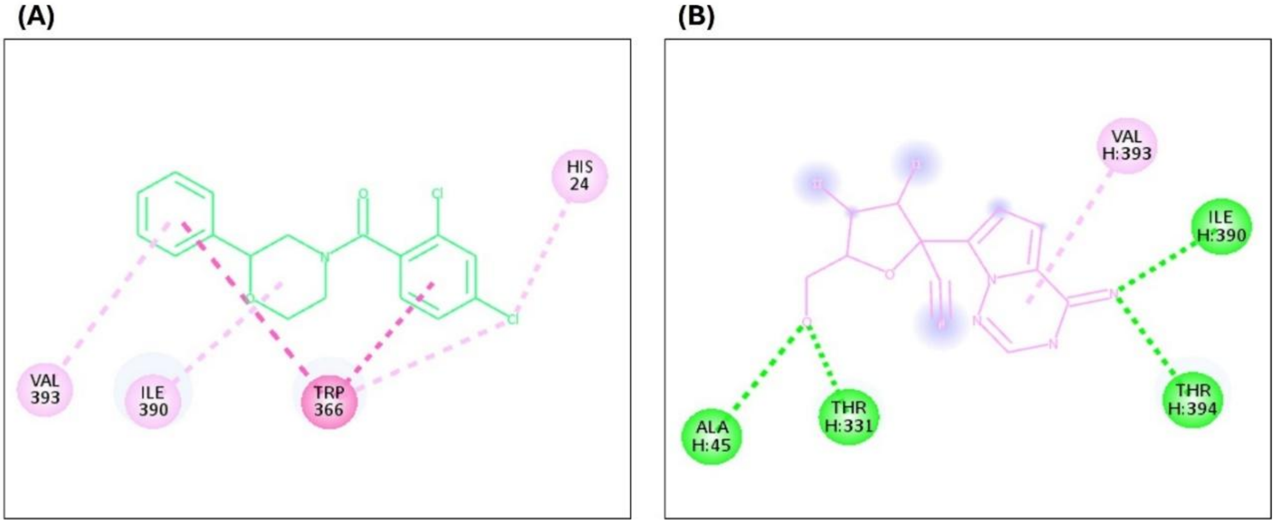

Figure S7. The 2D docked conformation of (A) F0045(S) (B) GS441524 in binding site of HA protein of H5N1.

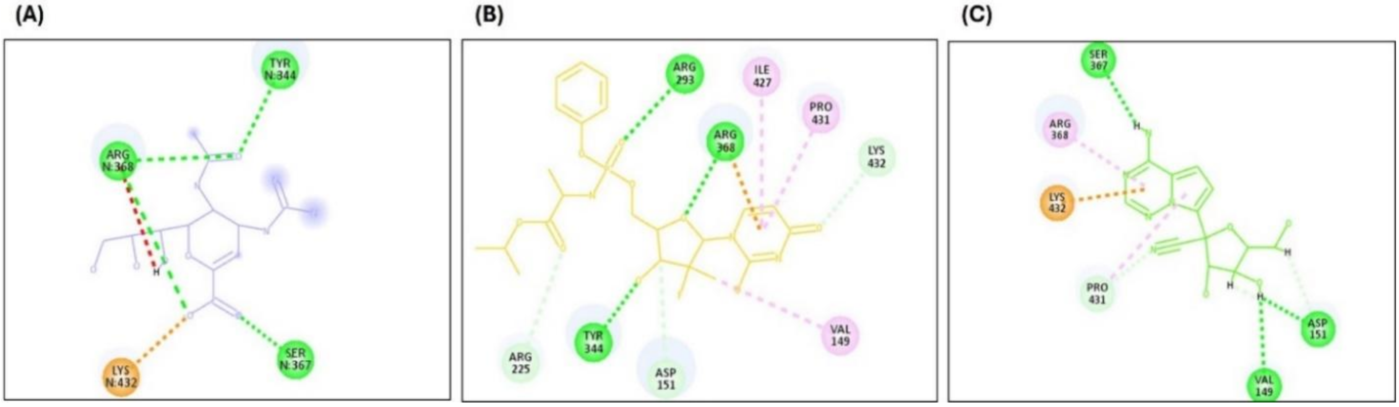

Figure S8. The 2D docked conformation of (A) Zanamivir (B) Sofosbuvir and (C) GS441524 in active site of NA protein of H5N1.

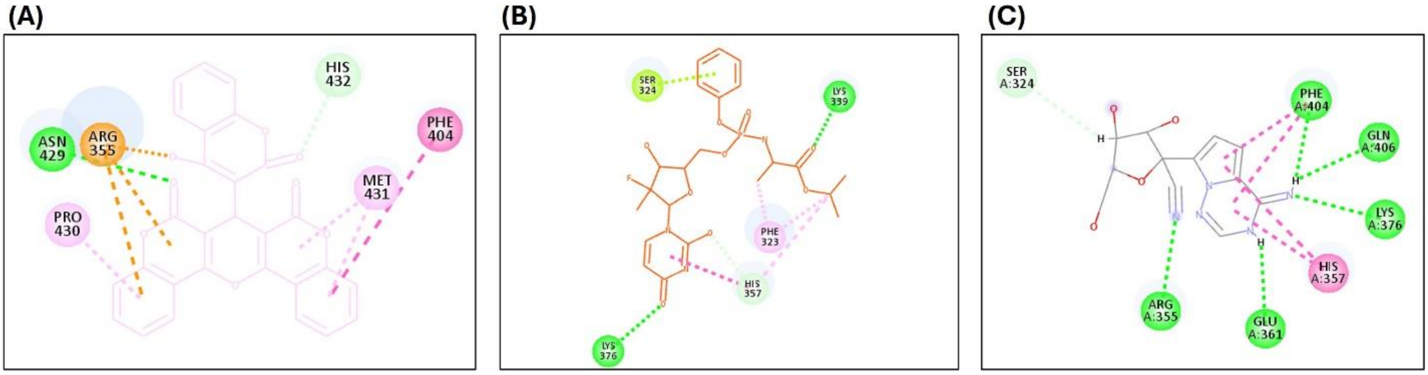

Figure S9. The 2D docked conformation of (A) PB2-39 (B) Sofosbuvir and (C) GS441524 in active site of PB2/CBD protein of H5N1.

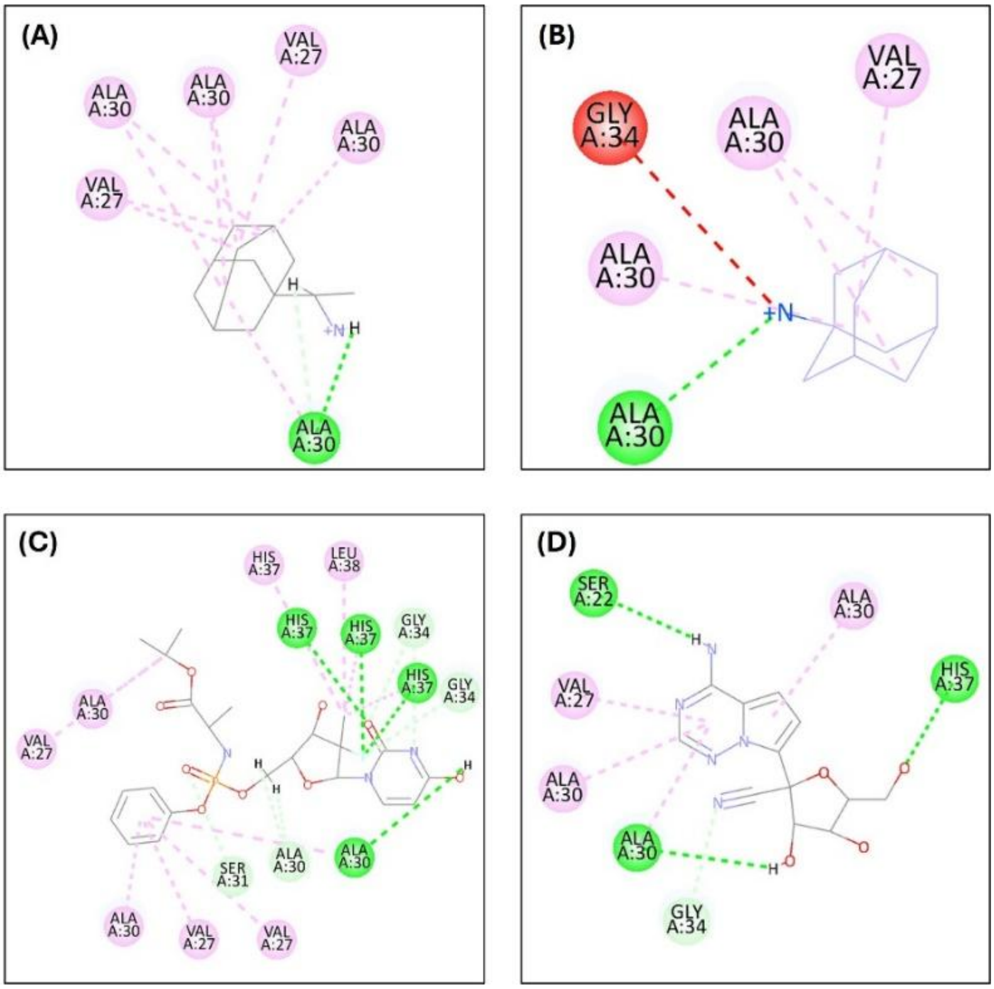

Figure S10. The 2D docked conformation of (A) Rimantadine (B) Amantadine (C) Sofosbuvir and (D) GS441524 in active site of M2 channel protein of H5N1.
